# Screening Preoperative Peptide Biomarkers for Predicting Postoperative Myocardial Infarction after Coronary Artery Bypass Grafting

**DOI:** 10.1371/journal.pone.0100149

**Published:** 2014-06-30

**Authors:** Zhibin Jiang, Ping Hu, Jianxin Liu, Dianjun Wang, Longyu Jin, Chao Hong

**Affiliations:** 1 Department of Cardiothoracic Surgery, The Third Xiangya Hospital, Central South University, Changsha, Hunan, P.R. China; 2 Department of Cardiothoracic Surgery, Xiangya Hospital, Central South University, Changsha, Hunan, P.R. China; University of Colorado Denver, United States of America

## Abstract

Postoperative myocardial infarction (PMI) is one of the most serious complications of cardiac surgeries. No preoperative biomarker is currently available for predicting PMI after cardiac surgeries. In the present study, we used a phage display peptide library to screen potential preoperative peptide biomarkers for predicting PMI after coronary artery bypass grafting (CABG) surgery. Twenty patients who developed PMI after CABG and 20 age-, sex-, and body mass index-matched patients without PMI after CABG were enrolled as a discovery cohort. Another 50 patients who developed PMI after CABG and 50 randomly selected patients without PMI after CABG were enrolled as a validation cohort to validate the potential peptide biomarkers identified in the discovery cohort. Fifty randomly selected healthy volunteers were also enrolled in the validation phase as a healthy control group. In the discovery/screening phase, 17 out of 20 randomly selected phage clones exhibited specific reaction with purified sera IgG from the PMI group, among which 11 came from the same phage clone with inserted peptide sequence GVIMVIAVSCVF (named PMI-1). In the validation phase, phage ELISA showed that serum IgG from 90% of patients in the PMI group had a positive reaction with PMI-1; in contrast, only 14% and 6% of patients in the non-PMI group and the healthy control group had a positive reaction with PMI-1, respectively. The sensitivity, specificity, positive predictive value, negative predictive value and accuracy of the PMI-1 phage clone to preoperatively identify patients who would develop PMI after CABG were 90.0%, 86.0%, 86.5, 89.5% and 88.0%, respectively. The absorbance value of the PMI-1 phage clone showed statistically significant correlation with the peak postoperative serum cardiac troponin I level (r = 0.349, *p* = 0.012) in the PMI group. In conclusion, we for the first time identified a mimic peptide (PMI-1) with high validity in preoperative prediction of PMI after CABG.

## Introduction

Postoperative myocardial infarction (PMI) is one of the most serious complications of cardiac surgeries, occurring in 3%–15% of patients [1]. Early mortality after PMI ranges from 3.5% to 25% [2]. PMI is also associated with reduced long-term survival and high morbidity and cost [3].

PMI can be due to incomplete revascularization of atherosclerotic vessels, technical problems at anastomotic sites, hemodynamic disturbances during and after surgery, and tachycardia [4]. The most common cause of PMI is imbalance between myocardial demand and supply [4]. Defining PMI is often difficult because most PMIs occur without symptoms in anesthetized or sedated patients, ECG changes are subtle and/or transient, and the creatine kinase-MB isoenzyme has limited sensitivity and specificity [5]. The recent universal definition of PMI is based on a rise and/or fall of cardiac biomarkers (preferably troponin) in the setting of myocardial ischemia: cardiac symptoms, ECG changes, or imaging findings [6]. Over 90% of troponin elevations began within 24 hours after cardiac surgery [2]. However, there is no biomarker currently available to predict preoperatively whether a patient would develop PMI after cardiac surgery. Discovering such biomarkers will allow us to optimize postoperative management for patients undergoing cardiac surgery by identifying those who need intensified postoperative surveillance. In addition, by predicting patients' susceptibility to PMI, the predictive biomarkers may also provide new insights into the molecular mechanisms underlying PMI pathogenesis.

Phage display technology is based on the ability to express foreign peptides as fusions to capsid proteins on the surface of filamentous M13-derived bacteriophage and was first described in 1985 by Smith et al. [7]. Surface display is achieved by inserting a peptide-encoding gene into the gene for a capsid structural protein. Billions of pooled peptides presented on phage particles form a phage display peptide library [7]. This technology was originally developed to map epitope-binding sites of antibodies by panning phage peptide libraries on immobilized immunoglobulins [8]. Since then, phage display has been widely used to screen targeting peptides in drug discovery and biomarker selection [8]–[10]. In the present study, we used a phage display peptide library to screen potential preoperative peptide biomarkers for predicting PMI after coronary artery bypass grafting (CABG) surgery.

## Materials and Methods

### Ethics Statement

This study was approved by the Ethics Committee of the Third Xiangya Hospital, Central South University. Written informed consent was obtained from all participants.

### Patients

From April 2011 to September 2013, 712 Chinese Han patients who underwent CABG with cardiopulmonary bypass (CPB) at the Department of Cardiothoracic Surgery of Xiangya Hospital and the Third Xiangya Hospital, Central South University were screened for PMI after CABG. Twenty patients who developed PMI after CABG and 20 age-, sex-, and body mass index (BMI)-matched patients without PMI after CABG were enrolled as a discovery cohort. Another 50 patients who developed PMI after CABG and 50 randomly selected patients without PMI after CABG were enrolled as a validation cohort to validate the potential peptide biomarkers identified in the discovery cohort. Fifty randomly selected healthy volunteers were also enrolled in the validation phase as a healthy control group. Patients with a history of renal failure, active liver disease, bleeding disorders or autoimmune diseases were excluded. Patients with a history of immunosuppressive therapy or a family history of coronary artery disease were also excluded. All antiplatelet agents were ceased at least 2 days before CABG in the patients. Intraoperative anesthetic, perfusion and cardioprotective management were standardized as follows: fentanyl/isoflurane anesthesia, nonpulsatile CPB (32°C to 35°C), crystalloid prime, pump flow rates >2.4 L/min per m^2^, cold blood cardioplegia, α-stat blood gas management, heparin to maintain activated clotting times >450 seconds, ε-aminocaproic acid infusion, and serial hematocrits kept ≥0.18 during CPB. Blood samples were collected from all screened patients (n = 712) at baseline (within 72 hours before CABG) and after CABG, and from all healthy volunteers (n = 50) within 72 hours after enrollment.

### Phage display reagents

The phage display peptide library and its host strain E.coli ER 2738 were purchased from New England Biolabs (Ipswich, MA, USA). The QlAprep Spin M13 kit was purchased from Qiagen (Valencia, CA, USA).

### Definition of PMI

PMI was defined as an increase of cardiac troponin I (cTnI) to greater than five times the 99th percentile of the normal reference range during the first 72 hours after CABG, together with the appearance of new pathological Q-waves or left bundle branch block, or angiographically documented new graft or native coronary artery occlusion, or imaging evidence of new loss of viable myocardium [11]. Blood samples were collected from all screened patients (n = 712) at baseline (within 72 hours before CABG) and at 1, 6, 12 and 24 hours after CABG. Measurement of serum cTnI was carried out using Siemens Immulite (Siemens Healthcare Diagnostics, Los Angeles, CA, USA) according to the manufacturer's recommended methods. The detection limit of the assay was 0.1 µg/L, and the 99th centile of the reference population was <1.0 µg/L. The 10% imprecision limit was 0.2 µg/L, and the upper measurement limit was 180 µg/L.

### Biopanning and amplification of phages

All phage-related procedures in this study were conducted as previously described [12]. Blood samples were allowed to clot and then centrifuged at 4000 rpm for 10 min. The serum was filtered with a micro-cell filter (Φ 0.22 µm) and then frozen at −70°C immediately. The serum pools were respectively obtained from patients with or without PMI and from healthy volunteers. Immunoglobulin G (IgG) from the serum pools was purified. To increase the screening specificity, microtiter wells were coated with purified sera IgG from patients without PMI to absorb non-specific phages from the phage peptide library, and the unbounded phages were then incubated with sera IgG from patients with PMI to screen for phages containing potential peptide biomarkers. Briefly, microtiter wells were coated overnight at 4°C with 100 µL of purified IgG (100 µg/mL) from patients without PMI. The plates were blocked with 3% nonfat milk for 2 hours at 37°C, and then washed 5 times with 0.05% Tween-20 in Tris-buffered saline. One hundred µL of diluted phage display library was added to the coated plates. After incubation for 1 hour at room temperature, the unbound phages were collected and added 100 µL/well into a plate coated with sera IgG from patients with PMI. After incubation for 1 hour at room temperature, the bound phages were eluted with 100 µL of 0.2-mol/L glycine-HCl (pH 2.2) and neutralized with 1 mol/L Tris-HCl (pH 9.1). The eluted phages were then amplified in the host strain and purified by precipitation for about 4 hours using 1/6 volume of polyethanol glycol/NaCl. Another two rounds of affinity selection were carried out in the same way, except that 1∶200 and 1∶400 sera dilution was added to 100 µL of diluted phages from the last round. The percentage of enrichment was calculated using the following formula: Enrichment of phage clones (%) = (eluted phages/added phages) ×100%.

### Phage enzyme-linked immunosorbent assay (phage ELISA)

Ten µL of eluted phages from the third round of biopanning were added to 200 µL of the host strain, cultured overnight, and incubated for 20 minutes at 37°C. The transformed host strain cells were then transferred to a culture tube with agarose, quickly vortexed and immediately poured onto a pre-warmed LB plate, and incubated overnight at 37°C. Twenty phage clones were randomly picked the next day. Each picked clone was amplified, purified and tittered. The ELISA wells were coated with 2×10^11^ phage particles and then blocked with 2% bovine serum albumin (BSA) in phosphate buffered saline (PBS). One hundred µL of a 1∶100 dilution of purified sera IgG from patients with PMI were subsequently added and allowed to incubate for 1 hour at 37°C. The wells were then washed 3 times with PBS containing 0.05% Tween-20. Horseradish peroxidase (HRP)-conjugated goat anti-human IgG antibody was added. Specifically bound antibodies were visualized by adding 3,3′,5,5′- tetramethylbenzidine (TMB), and the absorbance value at 450 nm was measured. With the original phage library as a negative control, phage clones were considered positive when the absorbance value was more than 2 times of that of the negative control. Positive phage clones in phage ELISA were precipitated with polyethanol glycol/NaCl. The phage DNA was extracted with the QIAprep Spin M13 kit and sequenced with an ABI PRISM 377 sequencer using the −96 g111 sequencing primer (5′-CCCTCATAGTTAGCGTAACG-3′) from the Phage Display Peptide Library kit.

### Determination of optimal reaction concentration

Chessboard titration was performed with diluted positive phage clones and sera IgG from patients with PMI to determine the optimal reaction concentrations for the positive phage clones and sera IgG from patients with PMI. The plates were coated with 100 µL of diluted phages overnight at 4°C, and then washed and blocked with 2% BSA. Diluted sera IgG were subsequently added and incubated for 1 hour at 37°C. After incubation with HRP-conjugated goat anti-human IgG antibody for 1 h at 37°C, TMB was added to each well, and the enzymatic reaction was allowed to proceed for 15 minutes at room temperature. The absorbance values were measured at 450 nm with a microplate reader. The original phage library was used as a negative control.

### Predictive validity of the positive phage clone

To evaluate the predictive validity of the positive phage clones, sera from 50 patients with PMI after CABG, 50 patients without PMI after CABG and 50 healthy volunteers were used to react with the positive phage clones using phage ELISA. With the original phage library used as a negative control, the phage ELISA results were considered positive when the absorbance value was more than 2 times of that of the negative control. Predictive validity indicators were calculated using the following formulas: sensitivity = true positive/(true positive+false negative); specificity = true negative/(true negative+false positive); positive predictive value (PPV) = true positive/(true positive+false positive); negative predictive value (NPV) = true negative/(true negative+false negative); accuracy = (true positive+true negative)/(true positive+false negative + true negative+false positive).

### Statistical analysis

Statistical analyses were performed using SPSS for Windows version 15.0. All continuous variables were expressed as mean±SD. Comparisons of means between two groups were performed with student t-tests. Comparisons of means among multiple groups were performed with one-way ANOVA followed by *post hoc* pairwise comparisons using Tukey's tests. Categorical variables were compared with Chi-square tests or Fisher's exact tests. A two-tailed *p*<0.05 was considered statistically significant in this study.

## Results

**Figure 1:**
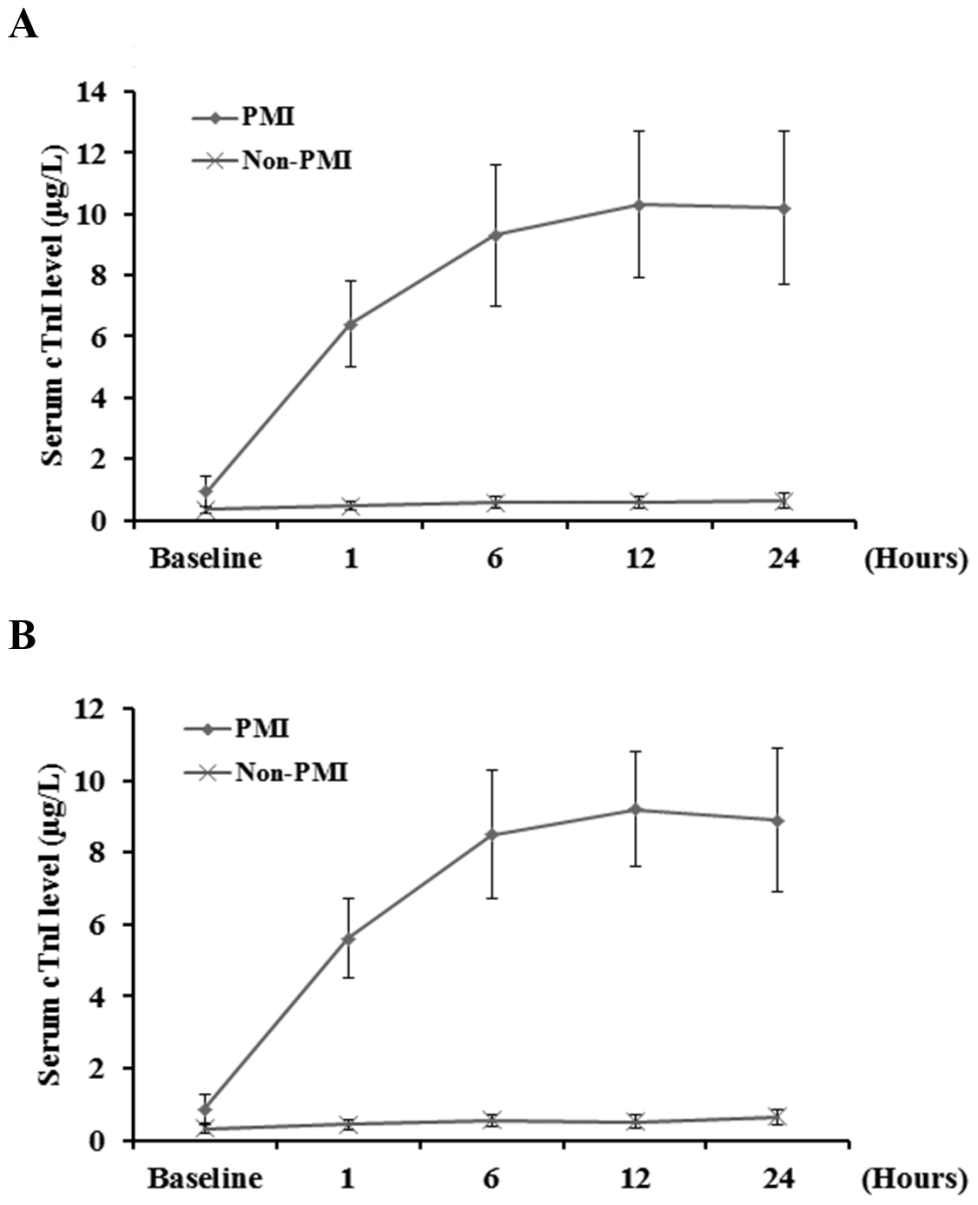
Serum cardiac troponin I (cTnI) levels after coronary artery bypass grafting (CABG) in the discovery and validation cohorts of patients. Serum cTnI levels were determined at baseline (within 72 hours before CABG) and at 1, 6, 12 and 24 hours after CABG in (A) the discovery (n = 20 each for the PMI and the non-PMI groups) and (B) the validation (n = 50 each for the PMI and the non-PMI groups) cohorts of patients. PMI, postoperative myocardial infarction.

cTnI, a contractile protein unique to heart muscle, is the gold standard for identifying MI [13]. In this study, PMI was defined as an increase of serum cTnI to greater than five times the 99th percentile of the normal reference range (<1.0 µg/L) during the first 72 hours after CABG, together with ECG changes or imaging findings. As shown in Fig. 1, in both the discovery (n = 20) and the validation (n = 50) cohorts, patients who developed PMI showed markedly increased serum cTnI levels within 24 hours after CABG, while the serum cTnI level remained low in patients without PMI. There was no significant difference in general characteristics (including age, sex, BMI, unstable angina, prior MI, hyperlipidemia, hypertension and diabetes mellitus) (Table 1) and disease characteristics (including the Gensini score and pre-CABG medications) (Table 2) between the PMI and the non-PMI groups at baseline in both the discovery and the validation cohorts. However, the PMI group had significantly longer duration of CPB and cross-clamp and higher postoperative mortality than the non-PMI group in both cohorts. In the validation phase, 50 healthy volunteers randomly selected within the age range of 50–76 years were also enrolled as a healthy control group, which had no significant difference in age (63.9±6.4 years) and sex (male, n = 33; 66%) compared with the PMI and the non-PMI groups in the validation cohort.

**Table 1:** Baseline general characteristics of the discovery and validation cohorts of patients.

Characteristics	Discovery cohort (n = 40)			Validation cohort (n = 100)		
	PMI (n = 20)	Non-PMI (n = 20)	*p*	PMI (n = 50)	Non-PMI (n = 50)	*p*
Age (years)	63.7±8.2	63.7±8.2	1.00	64.2±6.3	63.1±5.9	0.38
Male n(%)	16 (80)	16 (80)	1.00	35 (70)	32 (64)	0.67
BMI (kg/m^2^)	28.9±5.4	29.1±5.2	0.97	28.2±4.7	29.2±5.0	0.33
Unstable angina n(%)	14 (70)	12 (60)	0.74	26 (52)	30 (60)	0.55
Hyperlipidemia n(%)	18 (90)	19 (95)	1.00	42 (84)	45 (90)	0.55
Hypertension n(%)	15 (75)	14 (70)	1.00	34 (68)	31 (62)	0.68
Diabetes mellitus n(%)	9 (45)	6 (30)	0.51	19 (38)	13 (26)	0.28
Peripheral vascular disease n(%)	2 (10)	4 (20)	0.66	7 (14)	5 (10)	0.76
Prior MI n(%)	8 (40)	7 (35)	1.00	21 (42)	19 (38)	0.84
Prior Stroke n(%)	1 (5)	0 (0)	1.00	2 (4)	1 (2)	1.00
Current smoking n(%)	4 (20)	6 (30)	0.72	12 (24)	9 (18)	0.62
LVEF (%)	50.6±12.1	51.3±11.8	0.90	50.1±11.6	51.2±11.5	0.63
Preoperative hematocrit (%)	39.7±5.2	39.1±6.4	0.76	39.5±4.8	38.7±5.7	0.48

**Note**: Continuous variable values were expressed as Mean±SD. Categorical variables were expressed as n(%). BMI, body mass index; LVEF, left ventricular ejection fraction; PMI, postoperative myocardial infarction.

**Table 2:** Baseline disease characteristics and mortality outcomes of the discovery and validation cohorts of patients.

	Characteristics	Discovery cohort (n = 40)			Validation cohort (n = 100)		
		PMI (n = 20)	Non-PMI (n = 20)	p	PMI (n = 50)	Non-PMI (n = 50)	p
Disease pattern	LM or LM+1 vessel	4 (20)	5 (25)	1.00	9 (18)	9 (18)	1.00
	LM+2 or 3 vessel	5 (25)	3 (15)	0.70	12 (24)	6 (12)	0.19
	Two vessel	4 (20)	6 (30)	0.72	10 (20)	15 (30)	0.36
	Three vessel	7 (35)	6 (30)	1.00	19 (38)	20 (40)	1.00
	Gensini score	38.2±4.7	36.9±4.1	0.19	38.0±4.3	36.5±4.2	0.17
Pre-CABG medications	Aspirin	18 (90)	19 (95)	1.00	46 (92)	48 (96)	0.68
	Clopidogrel	4 (20)	2 (10)	0.66	8 (16)	7 (14)	1.00
	Statin	17 (85)	18 (90)	1.00	42 (84)	45 (90)	0.55
	β-Blocker	16 (80)	16 (20)	1.00	42 (84)	40 (80)	0.80
	ACE inhibitor/ARB	13 (65)	15 (75)	0.73	30 (60)	34 (68)	0.53
Procedural characteristics	No. of grafts	3.0±1.4	2.6±1.3	0.38	2.9±1.3	2.6±1.2	0.23
	Duration of CPB (min)	58±5	50±4	<0.01	57±4	47±3	<0.01
	Duration of cross-clamp (min)	37±4	32±3	<0.01	36±3	30±2	<0.01
Mortality outcomes	In-hospital mortality	1 (5)	0 (0)	1.00	3 (6)	1 (2)	0.62
	Total mortality 6 months after surgery	3 (15)	1 (5)	0.61	8 (16)	3 (6)	0.20

**Note**: Continuous variable values were expressed as Mean±SD. Categorical variables were expressed as n(%). ARB, angiotensin II receptor blocker; CPB, cardiopulmonary bypass; LM, left main; PMI, postoperative myocardial infarction.

**Figure 2:**
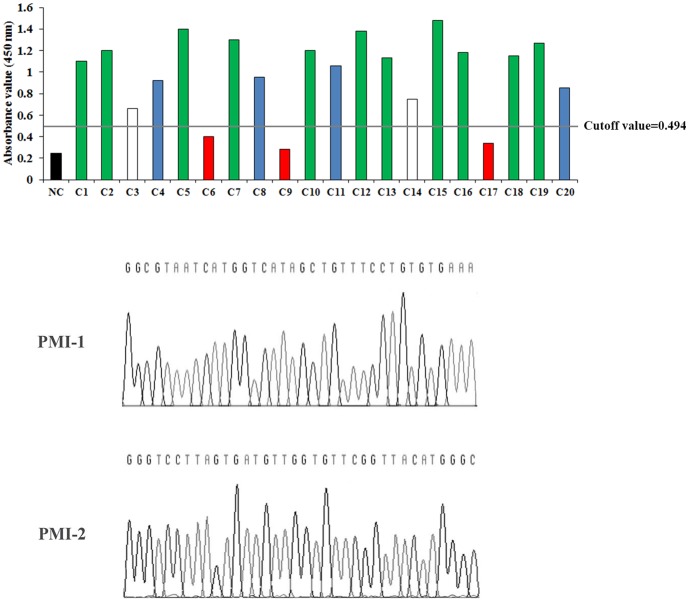
Inserted DNA sequence in positive peptide phage clones. After 3 rounds of biopanning, 20 peptide phage clones were randomly picked and reacted with sera IgG from patients with PMI after coronary artery bypass grafting. Phage clones were considered positive when their absorbance values in phage ELISA were above the cutoff value (0.494), which was set to 2 times of the absorbance value of the negative control (NC, black bar) at 450 nm. C1, C2, C5, C7, C10, C12, C13, C15, C16, C18 and C19 positive phage clones (green bars) had the same inserted DNA sequence 5′-GGC GTA ATC ATG GTC ATA GCT GTT TCC TGT GTG AAA-3′. The corresponding peptide sequence was GVIMVIAVSCVF (named PMI-1). C4, C8, C11 and C20 positive phage clones (blue bars) had the same inserted DNA sequence 5′-GGG TCC TTA GTG ATG TTG GTG TTC GGT TAC ATG GGC-3′. The corresponding peptide sequence was GSLVMLVFGYMG (named PMI-2). The negative phage clones were shown in red bars. The two single positive phage clones were shown in white bars.

As shown in Table 3, the percentage of phage increased from 2.5×10^−5^% to 2.3×10^−2^% after 3 rounds of biopanning, indicating a 1000 enrichment of phages that specifically bound to the sera IgG. As shown in Fig. 2, purified sera IgG from the PMI group at baseline (within 72 hours before CABG) had positive reaction with 17 out of 20 (85%) randomly picked phage clones. Among the 17 positive phage clones, 11 (C1, C2, C5, C7, C10, C12, C13, C15, C16, C18 and C19) had the same inserted DNA sequence 5′-GGC GTA ATC ATG GTC ATA GCT GTT TCC TGT GTG AAA-3′. The corresponding peptide sequence was GVIMVIAVSCVF (named PMI-1). Four other positive phage clones (C4, C8, C11 and C20) had the same inserted DNA sequence 5′-GGG TCC TTA GTG ATG TTG GTG TTC GGT TAC ATG GGC-3′. The corresponding peptide sequence was GSLVMLVFGYMG (named PMI-2). Using the NCBI Blast software, we searched for the identified peptide sequence in different protein databases including Swissprot and Protein Data Bank, and found that both PMI-1 and PMI-2 had no significant homology with other protein sequences (score <50). Chessboard titration was applied to determine the optimal reaction concentrations for the positive phage clones and sera IgG from the PMI group. The optimal coating concentrations were 10^11^ pfu/well and 10^12^ pfu/well for the PMI-1 and the PMI-2 phage clones, respectively. The optimal dilution of sera IgG from the PMI group was 1∶100 for both PMI-1 and PMI-2.

**Table 3:** Phage clone enrichment.

Biopanning	Phages Added (pfu/mL)	Phages Eluted (pfu/mL)	Enrichment (%)^a^
1	6.0×10^11^	1.5×10^5^	2.5×10^−5^
2	3.2×10^12^	1.9×10^8^	5.9×10^−3^
3	1.4×10^11^	3.2×10^7^	2.3×10^−2^

**Note**:

a Phage clone biopanning enrichment (%) = (eluted phages/added phages) ×100%.

To evaluate the predictive validity of PMI-1 and PMI-2, sera from the validation cohort at baseline (within 72 hours before CABG) and the healthy control group were used to react with the PMI-1 and the PMI-2 phage clones in phage ELISA, respectively. In the validation phase, phage ELISA showed that serum IgG from 90% of patients in the PMI group had a positive reaction with PMI-1; in contrast, only 14% and 6% of patients in the non-PMI group and the healthy control group had a positive reaction with PMI-1, respectively (Table 4). Although 96.0% of patients in the PMI group had a positive reaction with PMI-2, 52.0% of patients in the non-PMI group also showed a positive reaction (Table 4).

**Table 4:** Phage ELISA in healthy controls and the validation cohort of patients.

Positive phage clone	Experimental group	Absorbance Value	Positive number	Positive rate (%)
PMI-1	PMI (n = 50)	0.952±0.244^a^^,^ b	45	90.0^a^^,^ b
	Non-PMI (n = 50)	0.389±0.185^a^	7	14.0
	Healthy control (n = 50)	0.245±0.115	3	6.0
PMI-2	PMI (n = 50)	0.947±0.239^a^^,^ b	48	96.0^a^^,^ b
	Non-PMI (n = 50)	0.534±0.195^a^	26	52.0^a^
	Healthy control (n = 50)	0.240±0.121	2	4.0

**Note**: All continuous variables were expressed as Mean±SD. Comparisons of means among multiple groups were performed with one-way ANOVA followed by *post hoc* pairwise comparisons using Tukey's tests. Categorical variables were compared with Chi-square tests. PMI, postoperative myocardial infarction;

a *p*<0.05 vs. Healthy control;

b *p*<0.05 vs. Non-PMI.

As shown in Table 5, using the non-PMI group as a control, sensitivity of the PMI-1 and the PMI-2 phage clones to preoperatively identify patients who would develop PMI after CABG were 90.0% and 96.0%, specificity 86.0% and 48.0%, PPV 86.5% and 64.9%, NPV 89.5% and 92.3%, and accuracy 88.0% and 72.0%, respectively. Using the healthy control group as a control, sensitivity of the PMI-1 and the PMI-2 phage clones to preoperatively identify patients who would develop PMI after CABG were 90.0% and 96.0%, specificity 94.0% and 96.0%, PPV 93.8% and 96.0%, NPV 90.4% and 96.0%, and accuracy 92.0% and 96.0%, respectively.

**Table 5:** Predictive validity of PMI-1 and PMI-2.

Positive phage clone	Control group	Sensitivity (%)	Specificity (%)	PPV (%)	NPV (%)	Accuracy (%)
PMI-1	Non-PMI (n = 50)	90.0	86.0	86.5	89.6	88.0
	Healthy control (n = 50)	90.0	94.0	93.8	90.4	92.0
PMI-2	Non-PMI (n = 50)	96.0	48.0	64.9	92.3	72.0
	Healthy control (n = 50)	96.0	96.0	96.0	96.0	96.0

**Note**: All indicator values were expressed in percentage: sensitivity = true positive/(true positive+false negative); specificity = true negative/(true negative+false positive); positive predictive value (PPV) = true positive/(true positive+false positive); negative predictive value (NPV) = true negative/(true negative+false negative); accuracy = (true positive+true negative)/(true positive+false negative + true negative+false positive). PMI, postoperative myocardial infarction.

In the validation phase, the absorbance value of the PMI-1, but not the PMI-2 phage clone showed statistically significant correlation with the peak postoperative serum cTnI level (for PMI-1, r = 0.349, *p* = 0.012; for PMI-2, r = 0.254, *p* = 0.085) in the PMI group.

## Discussion

PMI is one of the most severe complications in patients undergoing cardiac surgery. Early diagnosis of PMI is important for optimal postoperative patient management [1]–[3]. However, PMI is a multifactorial disorder with significant inter-patient variability poorly predicted by clinical and procedural factors [1]. No preoperative biomarker is currently available for predicting PMI after cardiac surgeries. In this study, we for the first time identified a mimic peptide with high validity in predicting preoperatively whether a patient would develop PMI after CABG.

In the discovery/screening phase, the PMI group (n = 20) was matched with the non-PMI group in age, sex, and BMI to minimize background noise. In the validation phase, however, patients in the non-PMI group (n = 50) were randomly selected in order to test the predictive validity of the identified mimic peptides in a more realistic setting, which proved effective in revealing the low predictive validity of the PMI-2 mimic peptide (Tables 4 and 5). In this study, the PMI and the non-PMI groups in both the discovery and the validation cohorts showed significant difference in mortality outcomes and the duration of CPB and aortic cross-clamping, in agreement with previous studies [1], [2].

Phage display libraries are diverse collection of random peptides displayed on the surface of filamentous phages [7]. Phage display technology was originally developed to map epitope-binding sites of antibodies by panning phage peptide libraries on immobilized immunoglobulins [8]–[10]. It involves specific screening and affinity selection of a certain phage displaying peptide that is ligand for particular proteins. A major advantage of this technology is the extensive control of selection conditions, which allows preservation of the three-dimensional structure of peptides [14], [15]. Previous studies have used phage display peptide libraries to identify candidate biomarkers for diseases [8], [12]. Wang et al. identified a mimic peptide as a potential biomarker of ankylosing spondylitis using a phage display peptide library [8]. Weng et al. reported identification of a candidate biomarker for knee osteoarthritis by phage display [12]. In our study, the PMI-1 mimic peptide showed high validity in predicting preoperatively whether a patient would develop PMI after CABG. Patients who showed positive reaction with PMI-1 in phage ELISA had high chances of developing PMI after CABG (sensitivity 90%, PPV 86.5%, and accuracy 88.0%). Therefore, the PMI-1 mimic peptide could be used as a predictive biomarker to guide optimal postoperative management for patients undergoing CABG, for it will help surgeons preoperatively identify patients who will very likely develop PMI after CABG and thus need intensified postoperative surveillance. In addition, the phage ELISA assay used in this study is easy to perform and can be finished in less than 3 hours, which adds weight to the feasibility of using the PMI-1 mimic peptide as a biomarker. The clinical application value of PMI-1 in guiding optimal postoperative management of patients undergoing cardiac surgery will be explored in our future study with a larger patient sample.

cTnI is the gold standard for identifying PMI [13]. In our study, the absorbance value of the PMI-1 phage clone was found significantly correlated with the peak serum cTnI level after CABG. This suggests that PMI-1 could also be used to predict the severity of PMI after CABG, which will be examined in our future study with a larger patient sample. Since the PMI-1 mimic peptide showed no significant homology with other protein sequences, it is likely derived from an unknown protein. Exploration into the identity and functional roles of PMI-1 and its parent protein(s) may provide new insights into the molecular mechanisms underlying PMI pathogenesis.

In comparison to PMI-1, the PMI-2 mimic peptide showed low predictive validity (in particular, specificity, PPV and accuracy) in the validation phase, which was likely due to low-specificity binding between phages and sera IgG from the PMI group in the discovery/screening phase. This was in line with the screening data that in 20 randomly picked phage clones, only 4 clones with relatively low absorbance values expressed PMI-2, while 11 clones with relatively high absorbance values expressed PMI-1 (Fig. 2). Additionally, this was also supported by our findings that the absorbance value of the PMI-1, but not the PMI-2 phage clone showed statistically significant correlation with the peak postoperative serum cTnI level.

There are some limitations to this study: (1) We only enrolled patients undergoing CABG with CPB, for this was the predominant type of patients we could have for an adequate sample size. It will be interesting to find out the predictive validity of PMI-1 in patients undergoing off-pump CABG and other cardiac surgeries. (2) Only Chinese Han patients were enrolled in this study, which minimized background noise for the screening tests. Although Chinese Han accounts for 90% of the population in China and 19% of global population [16], enrollment of a single ethnicity in this study may limit the generalizability of our findings. Thus, it will be interesting to test the predictive validity of PMI-1 or screen for its counterparts in other ethnicities in future studies.

In conclusion, we identified a mimic peptide PMI-1 with high validity in preoperative prediction of PMI after CABG. The PMI-1 mimic peptide could be used as a predictive biomarker to guide optimal postoperative management for patients undergoing CABG. In addition, it may serve as a new basis for exploration into the molecular mechanisms underlying PMI pathogenesis.
